# Benchmarking Modern Day Pencil Beam Scanning Proton Therapy Treatment Times: Insights From Real Time Location Service Treatment Time Data

**DOI:** 10.1016/j.ijpt.2025.101208

**Published:** 2025-10-06

**Authors:** Kristen McConnell, Andrew Wroe, Maria Valladares, Lorrie LeGrand, Ulrich Ramos, Michael Chuong, Minesh Mehta, Alonso Gutierrez

**Affiliations:** 1 Department of Radiation Oncology, Miami Cancer Institute, Baptist Health South Florida, Miami, Florida, USA; 2 Department of Radiation Oncology, Herbert Wertheim College of Medicine, Florida International University, Miami, Florida, USA

**Keywords:** Proton therapy, Treatment delivery times

## Abstract

**Purpose:**

Collecting consistent and accurate treatment time data is challenging, particularly when relying on manual records or predictive models. Our center uses a Real-Time Location System (RTLS), which provides precise, automated tracking of patient movement throughout the clinic. This study leverages RTLS data to benchmark treatment times in a high-volume pencil beam scanning proton facility where ancillary systems like cone beam CT, surface imaging, and ultrasound bladder scans are used to aid in setup.

**Materials and Methods:**

We analyzed RTLS data from 12,551 fractions delivered between January 2023 and July 2024 across three gantries of an IBA Proteus Plus system. Treatment time was defined as the duration between a patient entering and exiting a gantry zone, recorded by infrared and radio frequency badges. Outliers and erroneous entries were excluded using clinical and statistical criteria. Mean and median treatment times were calculated by disease sites, subsites, and gantries. Imaging protocols and setup factors were provided to give context to the data.

**Results:**

Treatment times ranged from 26 minutes (vertebrae) to 52 minutes (craniospinal and gynecologic), with an overall average of 35.5 minutes. High-volume sites such as prostate (4479 fractions), head and neck (2153), and central nervous system (CNS) (1954) showed relatively tight distributions, but had many outliers due to complex setups or anatomical variability. Discrepancies in treatment time across gantries were observed, with certain gantries consistently faster, likely due to staff familiarity and case concentration on specific gantries.

**Conclusion:**

RTLS provides a scalable method for tracking treatment times in proton therapy. These data offer an unbiased, data-driven foundation for clinical and business planners to develop more accurate and sustainable proton center models as well as serve as benchmark data for other proton centers seeking to optimize performance.

## Introduction

The modern proton therapy center is a complex environment where numerous factors influence the total time required to treat a patient. Centers today may utilize multiple or single gantry solutions, advanced imaging technologies like kV-cone beam CT (kV-CBCT) for target localization, surface imaging systems for patient pose setup, and ultrasound bladder scans to verify bladder volumes, among other tools. Collecting consistent and accurate treatment time data in such a multifaceted environment is a significant challenge for many centers.

One approach is to extract appointment start and end times from the record-and-verify system; however, this method is prone to inaccuracies since some timestamps may rely on manual input. Another approach is to use predictive models based on parameters such as layers, spots, beams, and other treatment components.^1^, ^2^ These models, while potentially effective, require continual updates to remain relevant as technology evolves and do not account for factors associated with patient setup.

At our center, we employ a system called Real Time Location Service (RTLS) (Midmark Corporation, Versailles, USA), which provides a reliable method of tracking treatment times. Proton therapy patients receive a badge each day at check-in, equipped with a receiver that tracks ingress and egress timestamps as they move through designated zones within the clinic. Unlike manual input-based systems, RTLS provides precise and dynamic real-time tracking that accounts for factors such technological advancements, staff learning curves, machine downtime, and novel tools implemented for treatment or setup. The RTLS system offers a robust method for measuring treatment times in a way that adapts to real-world operational changes.

There are many useful applications of the RTLS system in general healthcare^3^, ^4^, ^5^, ^6^, ^7^, ^8^, ^9^, ^10^, ^11^ including some in radiation oncology. However, to our knowledge, no published work has utilized RTLS to track treatment times in a pencil beam scanning proton therapy treatment facility. This is particularly relevant given the rapid growth of proton therapy centers worldwide. In the past 5 years, at least 29 new centers have opened.^12^ The National Association of Proton Therapy (NAPT) reports that the number of patients treated with protons nearly tripled between 2012 and 2021.^13^ With this expansion, there is a pressing need for data from modern centers to inform operational planning for new facilities and provide benchmarks for existing ones.

With this in mind, we collected treatment time data from January 2023 to July 2024. These data are presented in several formats: average and median times per treatment site and subsites across the study period as well as variations in treatment times among our gantries for each clinical site. Additionally, we provide information on the standard imaging protocols for each treatment site at our institution to offer further context for potential underlying factors influencing treatment times.

## Methods

Our center operates a 3-gantry IBA Proteus Plus system, with all treatment rooms constructed in an identical fashion including beam matching to allow ease of patient transfer and schedule balancing. Each of the 3 treatment rooms feature 360° gantries, pencil beam scanning with a 30 × 40 cm^2^ field size, cone-beam computed tomography (CBCT), C-RAD surface imaging (CRAD AB, Uppsala Sweden), 6°-of-freedom (6DOF) Leoni couch, and kVue based table and immobilization system (CQ Medical Kalona, IA). The clinic treats a wide range of clinical cases including comprehensive breast (reconstructed breast expanders and chest wall), prostate, craniospinal irradiation (CSI), brain, lung, head and neck, and mediastinum. Advanced and/or time-consuming techniques such as intensity modulated proton therapy (IMPT), GRID therapy, pulsed reduced dose rate therapy, gated breath hold treatments, craniospinal therapy, and treatments under anesthesia are routinely used.

### Real-time location system (RTLS)

The RTLS system (Midmark RTLS) is a commercially available system that is customized to each client’s needs. The system was installed to meet the needs of our center and had customizations completed for the data collection metrics to ensure they would be useful to the center. Since the inception of the proton center, RTLS has been in place to monitor the ingress and egress of patients and equipment within the facility. The system has 3 main components: wearable badges (detectors), sensors, and software. Patients and staff wear badges with unique IDs, which transmit infrared (IR) signals and backup radiofrequency (RF) signals. Sensors located at key points throughout the facility detect these signals and record precise timestamps.

**Figure 1:**
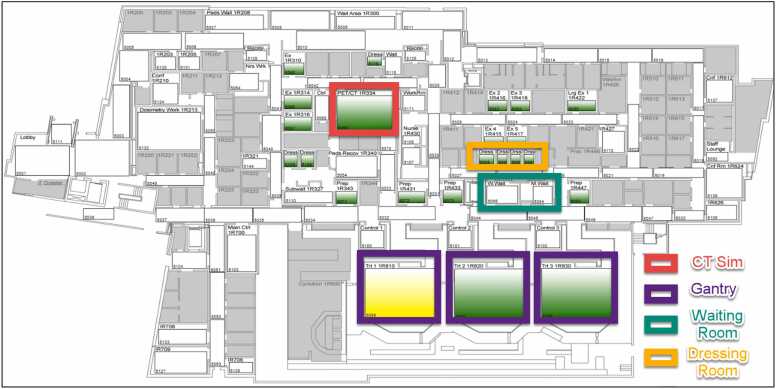
Diagram of the proton clinic RTLS sensors/zones. The colors indicate room status (green for available and yellow for occupied). **Abbreviation:** RTLS, real time location service.

For this clinic, the RTLS setup includes 143 sensors strategically placed in zones such as exams room, waiting areas, dressing rooms, and proton gantries. Figure 1 illustrates the proton clinic layout, with *Trt 1 1R810*, *Trt 2 1R820,* and *Trt 3 1R830* corresponding to Gantry 1, 2, and 3, respectively. Zone status is visually indicated: green for available and yellow for occupied. The RTLS tracks multiple time metrics, including waiting-to-treatment time, waiting-to-exam time, time alone in the exam room, time spent with medical staff, and treatment times. For this study, the primary focus was on treatment time, defined as the duration from when a patient’s badge pings the gantry receiver to when it pings a receiver in the waiting room, dressing room, lobby, or exam room.

### Data collection

For this project, we collected and analyzed data between January 2023 to July 2024 which represented a stable operational period for the center. At this time, all 3 gantries were fully operational, staff were trained in best practices, practice standards were defined, codified, and operationalized, staffing levels were stable, and compliance with RTLS badge usage was consistent. Using the RTLS system, treatment times were recorded for 12,551 fractions across various treatment sites. Each fraction was categorized by treatment site and subsite.

### Data processing

To ensure accuracy, preprocessing was performed to exclude outliers and errors. Treatment times exceeding 3 hours were removed, as these likely resulted from patients forgetting their badge in the treatment room or incorrect location. Times under 15 minutes were also excluded, as they likely represented cases of bladder fill issues requiring the premature removal of patients from the treatment room, setup interruptions, or machine downtime. Outliers beyond the mean ± 3 standard deviations were removed as values beyond this range are statistically rare. Descriptive statistics were then calculated for each treatment site and subsite including average and median times over the entire study period. Time variations among gantries and disease sites were charted.

### Technology used in clinic

Each of the 3 gantries is beam-matched, capable of full 360° treatment angle rotation, and couch rotation from 270° to 90° (180° of travel capability). Each room is equipped with identical imaging systems, including 2 orthogonal kV imagers (Rad A and Rad B) and CBCT capabilities using Rad B (orthogonal to beam). Imaging panels are mounted on the gantry and rotate with it, and they are retractably inserted into the treatment room from the back wall. CBCT protocols include head (small and medium field-of-view [FOV]), thorax (medium FOV, medium high speed FOV), breast (medium and large FOV), and pelvis (medium and large FOV) options, with scan times ranging from 0.5–2 minutes depending on half/full gantry rotation and rotation speed (3°/second for regular speed and 6°/second for high speed).

Each room also features a 3-camera C-RAD surface imaging system for patient setup and monitoring. Additionally, a US bladder scanner is available for pre-treatment bladder volume measurements. The kVue One Proton Couch Top (CQ Medical Kalona, IA) is rigidly mounted on the Leoni positioner, with 3 interchangeable inserts for various setups. Compression belts and the SDX System are also utilized for motion management. The center uses ARIA (Varian Medical, Palo Alto, CA) as the record and verify system, but the IBA AdaptDeliver and AdaptInsight provide the treatment delivery and imaging controls software, respectively.

### Prescription imaging protocols used in clinic

The typical treatment workflow begins with orthogonal setup fields selected based on the treatment angle’s proximity to cardinal angles. CBCT is performed if prescribed by the physician. For breast cases and select other cases, the C-RAD surface imaging system is used to aid in adjusting patient position during setup before imaging. For efficiency, the treatments are arranged to proceed counterclockwise with couch kicks at the end of the fields. Couch kicks must be imaged to verify table movement; however, shifts are not made from these port images. If observed shifts are larger than 2 mm or 1°, the therapists return to setup and re-image and shift. Additionally, if the couch kick is not able to be imaged, C-RAD is used to track. Generally, outside of couch kicks, we do not have cases that require mid-treatment imaging unless the patient has been suspected to have moved or the gantry has been down for longer than 15 minutes. In that case, a set of verification images or CBCT would be taken to ensure the positioning has not changed.

When CBCT images are sent from IBA AdaptInsight to ARIA, they include registration data, but kV images do not include the registration. To address this, post-shift kV images are acquired and sent to ARIA for offline review. Although this adds extra imaging time, it is necessary to ensure images in the treatment position are available in offline review. Image guidance protocols are specific for disease sites and subsites and influence treatment duration. To contextualize time data, Table 1 provides a summary overview of the image-guide radiotherapy (IGRT) guidelines used at our institution. Prostate and prostate bed IGRT workflows are the only to utilize post-CBCT kV imaging. For these cases, the CBCT is utilized to assess tissue differences in the beam path while the post-CBCT kV images quantify any fiducial migration due to bladder fill during the positioning process.

**Table 1:** IGRT guidelines per site for proton.

**Site**	**Treatment workflow**
Abdomen	kV → Apply Shifts → CBCT → Send CBCT Images → Treat
Brain	Two different workflows depending on prescription: 1. CBCT → Apply Shifts → Send CBCT Images → Treat 2. kV → Apply Shifts → KV → Send Clean kV Set → Treat
Breast(Regular or expander)	Two different workflows depending on prescription: 1. C-Rad → kV → Apply Shifts → CBCT → Apply Shifts → Send CBCT → Treat 2. C-Rad for leveling → kV → Apply Shifts → kV → Send Clean kV Set → Treat
H&N	kV → Apply Shifts → CBCT → Send CBCT Images → Treat
Pelvis(Non Prostate)	kV → Apply Shifts → CBCT → Send CBCT Images → Treat
Prostate	Two different workflows depending on prescription: 1. kV → Apply Shifts → kV → Send Clean kV Set → Treat 2. kV → Apply Shifts → CBCT → Send CBCT → kV → Send Clean kV → Treat (CBCT once per week)
Prostate Bed	kV → Apply Shifts → CBCT → Send CBCT → kV → Send Clean kV → Treat
SDX	kV → Apply Shifts → CBCT → Send CBCT Images → Treat
Thorax	kV → Apply Shifts → CBCT → Send CBCT Images → Treat

All couch kicks need kV imaging OR CRAD, if kV imaging not possible.

## Results

From January 2023 to July 2024, 14,025 fractions were collected and after pre-processing, 12,551 fractions were analyzed. Table 2 shows the mean and median treatment times by disease site, as well as the numbers of fractions for each site. Treatment times range from 26 minutes (vertebrae) to 52 minutes (craniospinal and gynecological). The prostate site has the highest number of fractions, at 4479, followed by head and neck with 2153 fractions, and central nervous system with 1954 fractions. The overall average for all the treatment sites is 35.5 minutes. Many of the site-specific treatment time averages are in a ±5 minutes range around the overall average. Bones, Breast, Prostate and Vertebrae are all more than 5 minutes under the overall average, while CSI and gynecologic (GYN) are more than 5 minutes over the overall average.

**Table 2:** Mean/median treatment time with standard deviation and fractions per treatment site.

**Site**	**Mean (min)**	**Median (min)**	**Delivered fractions**	**Percentage of total delivered fractions**
Vertebrae	26 ± 7	24	99	0.8%
Prostate	29 ± 11	27	4479	35.7%
Bones	30 ± 8	29	90	0.7%
Breast	30 ± 11	28	1957	15.6%
Mediastinum	32 ± 12	30	428	3.4%
Abdomen	32 ± 14	32	162	1.3%
CNS	33 ± 14	28	1954	15.6%
Extremity	34 ± 9	32	22	0.2%
Esophagus	35 ± 14	34	381	3.0%
Lung	36 ± 14	33	563	4.5%
Anal/Rectum	36 ± 15	34	98	0.8%
HN	37 ± 12	35	2153	17.2%
Liver	40 ± 12	33	72	0.6%
GYN	51 ± 16	47	34	0.3%
CSI	52 ± 19	48	60	0.5%

CNS, central nervous system; CSI, craniospinal irradiation; GYN, gynecologic.

**Figure 2:**
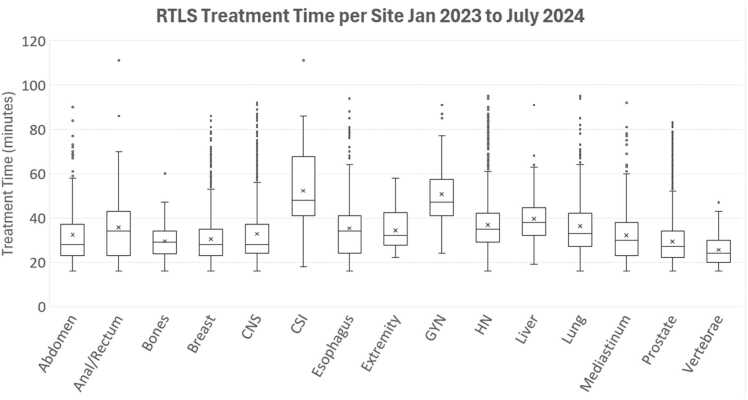
Box and whisker plot of treatment times per clinical site for January 2023 to July 2024.

Figure 2 shows the box-and-whisker plot comparing treatment times (in minutes) across various clinical sites. Craniospinal treatments exhibit the longest median treatment time and the widest range, indicative of the complexity of treatment and setup as well as the need for anesthesia in pediatric cases and the palliative nature of the urgent CSI cases. The extremity, prostate, and vertebrae cases have shorter median times as well narrower distributions, indicative of more predictable and consistent treatment durations. Breast and lung also have relatively lower median values, but have wider ranges, potentially linked to patient specific or setup related differences.

**Figure 3:**
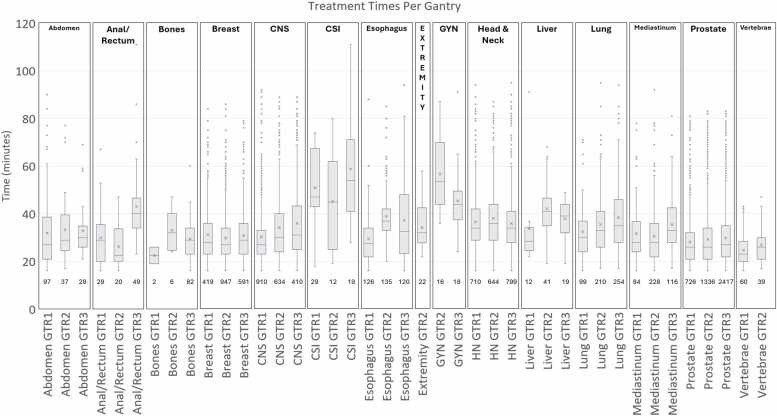
Box and whisker plot of treatment times per clinical site categorized by gantry for January 2023 to July 2024.

Figure 3 shows the box-and-whisker plot comparing treatment times (in minutes) across multiple gantries (GTR1, GTR2, GTR3) for various clinical sites. The number of fractions considered for each subgroup is shown under the box-and-whisker plot. The craniospinal treatments exhibit a notably larger range and higher median treatment times compared to others with GTR1 showing the lowest median treatment time. GTR1 also has the most fractions delivered for CSI, almost double the amount of each other gantry. Not all clinical sites show variation amongst gantries. Breast, head and neck, and prostate show fairly uniform times across the gantries. For breast GTR2 has double the amount of fractions delivered as GTR1 and GTR3. Head and neck have a very similar amount of fractions delivered across each gantry. And for prostate, GTR3 has double the amount of fractions delivered as compared to GTR1 and GTR2. For anal/rectum, GTR1/2 show similar times while GTR3 is elevated. GTR3 also has double the number of fractions delivered compared to GTR1 and GTR2. For many sites, GTR1 appears to be the fastest gantry with GTR3 appearing to be the slower. There are also many points that sit outside the whiskers, indicating outliers.

Table 3 shows a detailed breakdown of treatment times and counts for clinical sites and subsites. For Abdomen, the mean treatment time is 35 minutes. For the Anal/Rectum, the average was 35 minutes. The rectum had the shorter average time at 26 minutes while the anal canal and anal cases represented most of the treatment and averaged 37 minutes. For breast cases, most cases are close to the average of 30 minutes, while the right breast showed a large difference at 51 minutes. However, that was only associated with 9 fractions. For CNS, the majority of fractions are for brain and exhibited a 30-minute average. For CSI, this treatment stands out with the highest mean treatment time at 55 minutes. The pediatric CSI and urgent CSI cases tended to be shorter (38 and 44 minutes). For head and neck, there is wide variation in the average time across subsites. Oropharynx accounts for the largest portion of treatments at 1039 or 48% of the treated head and neck fractions. For lung, there were small variations in the subsite, but all were within ±4 minutes of the mean. For prostate, cases with nodes appear to take longer than prostate only cases.

**Table 3:** Treatment times per sub-category clinical site.

Site	Sub-site	Mean±σ (min)	Count
*Abdomen* Mean/Median = 35/32 min 225 fractions	Abdomen	33 ± 16	87
	Pancreas	30 ± 13	48
	Retroperitoneal LN	35 ± 12	27
*Anal/Rectum* Mean/Median = 35/34 min 98 fractions	Anus/Anal Canal	37 ± 15	90
	Rectum	26 ± 12	8
*Bones*	Bones	30 ± 8	90
*Breast* Mean/Median = 30/28 min 1957 fractions	Axilla (Ipsilateral)	25 ± 9	82
	Breast (Ipsilateral)	29 ± 12	157
	Breast (Bilateral)	38 ± 10	48
	Breast + Nodes (Ipsilateral)	30 ± 11	249
	Breast + Nodes (Bilateral)	31 ± 7	15
	Chest wall (Ipsilateral)	35 ± 10	83
	Chest wall + Nodes (Ipsilateral)	30 ± 10	1164
	Chest wall + Nodes (Bilateral)	37 ± 10	80
	Nodes	36 ± 12	79
*CNS* Mean/Median = 33/28 min 1954 fractions	Base of Skull	31 ± 12	215
	Brain	33 ± 14	1714
	Pituitary	27 ± 9	25
*CSI* Mean/Median = 52/48 min 60 fractions	CSI	55 ± 18	46
	Urgent CSI	44 ± 17	10
	Peds CSI	38 ± 25	4
*Esophagus*	Esophagus	35 ± 14	381
*Extremity*	Extremity	34 ± 9	22
*Gynecological*	Gynecological	52 ± 16	34
*Head and Neck* Mean/Median = 37/35 min 2153 fractions	Base of Tongue	37 ± 13	12
	Glands (Ipsilateral)	32 ± 11	323
	Maxilla	32 ± 10	44
	Nasopharynx	43 ± 12	218
	Neck (Ipsilateral)	35 ± 12	160
	Oropharynx	37 ± 12	1039
	Sinus	36 ± 12	71
	Thyroid	41± 10	43
	Misc (nose, cheek, mandible, larynx, etc)	36 ± 12	243
*Liver*	Liver	40 ± 12	12
*Lung* Mean/Median = 36/33 min 563 fractions	Lung, Left or Right	36 ± 13	504
	Lungs, Bilat	40 ± 16	59
*Mediastinum*	Mediastinum	32 ± 12	428
*Prostate* Mean/Median = 29/27 min 4479 fractions	Pelvis	38 ± 13	157
	Prostate (or +SV, +SVprox) Only	26 ± 11	1169
	Prostate + SV + Nodes	30 ± 11	1956
	Prostate Bed	31 ± 11	1143
	Prostate Bed + Nodes	33 ± 9	54
*Vertebrae*	Vertebrae	26 ± 7	99

CNS, central nervous system; CSI, craniospinal irradiation.

## Discussion

The prostate + SV + nodes (1947), brain (1714), prostate bed (1143), oropharynx (1039), and left-sided chest wall + lymph nodes + IMN (984) accounted for 55% of the treated 12,551 fractions. These high-volume sites represent a significant opportunity for improving efficiency, as even modest reductions in treatment time could lead to meaningful operational gains. Notably, box-and-whisker plots revealed that the brain, HN, prostate, and breast sites exhibit a high number of outliers, representing treatments that exceed average times.

For the brain, these outliers likely stem from additional setup complexities such as multiple couch adjustments or dose rate reductions introduced by increased time on table. In HN cases, anatomical changes often necessitate physician and physicist intervention, contributing to longer times. Prostate treatments, while among the most optimized workflows with a mean time of 29 minutes, face variability due to bladder or rectal filling, which is checked inside the treatment room. For breast treatments, challenges such as shifting tissue expanders and setup difficulties lead to extended time for some cases. Addressing these sources of variability through targeted process improvements could enhance overall efficiency. Areas of high anatomical change such as lung and HN get pre-scheduled verification CTs (or quality assurance CTs [QACTs]), which allows meaningful changes to be identified and replanned to improve quality and prevent long term issues with setup. Other sites used to have regularly scheduled verification CTs as well, but based on our clinical experience, these have been eliminated and are checked on an as needed basis. To facilitate real-time evaluation of the online CBCT, the center has implemented IGRT evaluation structures on a per patient basis. One example is the 10% isodose line, which is used to quickly focus the assessment of tissue differences to areas in the beam path and facilitate the decision-making process of performing an ad hoc verification QACT.

The data also revealed discrepancies in treatment times across gantries for many clinical sites. For example, patients treated on different gantries for the same site may experience inconsistent treatment durations, which could impact patient satisfaction. Research by Lamba et al suggests a correlation between patient anxiety/perceived pain and longer waiting times.^14^ A key driver of these differences could be staff assignment practices, as RTTs are typically assigned to a single gantry for extended periods, potentially limiting the cross-training and consistency. Introducing a rotation-based staffing model and enhanced training across gantries could help minimize these discrepancies. Additionally, the concentration of certain cases, such as CSI treatments on GTR1, naturally results in longer times on other gantries (GTR2 and GTR3) where these cases are less common. Adjusting workflows or redistributing specific cases across gantries could further improve efficiency.

Sites with lower counts, such as extremities (22 fractions) and liver (9 fractions), demonstrated treatment times close to the overall average. This highlights the adaptability of existing protocols for less common cases. Prostate treatments, which accounted for the highest fraction count (4479 fractions), maintained the shortest mean treatment time (29 minutes) among major sites, in spite of the fact that a substantial majority of these patients also receive pelvic nodal radiotherapy since we predominantly triage high-risk prostate cancer patients for proton therapy. This reflects the well-optimized protocols and routine workflows for this site, though bladder filling remains a recurring factor in alignment variability.

When comparing our center’s times to other reported studies, notable differences emerge. For example, another group reported mean treatment times of 11.5 minutes for non-respiratory synchrony cases and 30.1 minutes for pediatric sedation cases.^15^ Our corresponding lung and pediatric CSI times were 36 and 38 minutes, respectively. While our times are higher, they reflect a larger sample size and a site-specific categorization approach rather than a broad grouping by patient population. All the patients in our lung dataset were treated either by abdominal compression or free breathing. Another study which used in-line scanning reported average times for brain (34 minutes), HN (29.6 minutes), lung (46.6 minutes), liver (48.1 minutes), abdomen (31.5 minutes), Prostate (31.6 minutes) and CSI (60 minutes).^2^ At our center, brain and prostate times were similar (33 minutes and 29 minutes), but HN, lung, and liver were slower by 7, 10, and 13 minutes, respectively, while CSI was faster by 8 minutes. These differences are likely attributable to variations in IGRT practices and treatment philosophies, as shown in Suzuki et al., where treatment time increased quadratically with the number of fields used.^1^ For example, some centers do not treat both opposed lateral fields daily for prostate, significantly reducing treatment time compared to our approach. For our center, cases represented in the prostate, breast, esophagus, and rectum data set are 2–3 field arrangements. Our HN cases generally utilize 3–6 fields, GYN 2–5 fields depending on inguinal involvement, lung 2–3 fields, mediastinum 3 fields, CNS 3–4 fields, and CSI 3–4 fields. While we did not correlate treatment time directly with number of beams, it can be observed that cases like anal/rectum and esophagus took a very similar amount of time to HN where the number of beams is quite different. This indicates that the number of fields may not be the largest driver in our center for the total treatment time. There are other factors that influence the time such as technical planning parameters (spot spacing, layer spacing, etc), patient and case specific factors (bladder and rectal filling), selected image guidance (kV vs CBCT) and in-room efficiency items (field order optimization to reduce unnecessary gantry rotations, indexed immobilization, use of skin marks or C-RAD for setup, etc.).

Since our center has 3 gantries and shares one cyclotron, only one gantry can deliver beam at one time. This means if rooms are requesting at similar times, there is a queue. The treatment times data presented here is inclusive of that time. The data reported by the RTLS system unfortunately cannot provide meaningful information on the efficiency of the queue. Using the RTLS data, there is no way to know if rooms were in the queue together. That being said, this data is representative of a typical day in our center and if there is waiting time in the queue, it is included and helps to show us what is realistic. Additionally, our beam-on times range from 30 seconds to about 4 minutes. Our layer switch time is 0.8 seconds, and, in the worst-case scenario of a patient being third in queue, this would result in an 8-minute wait. This is also not systematic delay as patient’s appointment spots change day to day. We also incorporate strategies into our planning to keep beam-on times reasonable. For example, in cases that utilize the SDX system for motion management, we optimized our layer and spot spacing to balance quality with treatment time. Our longest treatment times are generally observed with the longest fields like CSI, prostate and nodes, and GYN. Hypofractionated cases like accelerated partial breast irradiation, while have high dose per fraction, are still smaller fields. In these low fraction, high dose cases, our beam-on times are generally on the lower side of our time range.

When considering efficiency at a proton center, there is also the question of unused room time for things such as downtime, equipment changes, nozzle changes, and cleaning among many items. Our center has a greater than 98% uptime and does not require nozzle swaps. Any COVID+ patients are treated at the end of the day, so that the cleaning extends after treatment is completed in that room.

Finally, we note that inter- and intra-patient variability may be contributors to treatment time distributions. A detailed evaluation of variability within and across patients for each disease site could provide additional insights into workflow optimization. However, such an analysis requires patient-level stratification and integration with clinical records, which will likely require a larger patient dataset for each disease site. Future investigation is warranted and will serve to complement the benchmarking data presented here.

The RTLS system has been instrumental in identifying practice standards within the center and providing per-site data on average treatment time slots. These insights support efficiency improvements by enabling the pre-population of time slots based on disease site, which can be actively monitored and adjusted as needed. However, limitations exist, including inconsistent badge usage, the effect of learning curves due to new staff onboarding, and the inclusion of wait times embedded in a shared beam facility. For example, inpatient CSI cases and pediatric anesthesia treatments often lack RTLS badges, making their true counts underrepresented in the data. These limitations emphasize the importance of refining data collection processes to improve the accuracy and utility of future analyses.

Lastly, there have been some improvements in our clinical practice due to the RTLS data. We have used this data as a catalyst to critically review our in-room workflows and compare them to established best practices. The RTT leadership used this data to identify improvements to efficiency such as revamping the initial patient setup, removing redundant imaging, and improving staff training and competencies. In recent months, we’ve seen treatment times reduce in CNS by 5 minutes (15% decrease), head and neck by 10 minutes (27% improvement), CSI by 18 minutes (34% reduction), and breast by 5 minutes (16% reduction). Overall, this totals to approximately 1-2 hours per day of time savings in the proton clinical operations.

## Conclusion

Between January 2023 and July 2024, our center delivered 12,551 fractions with a high level of performance and uptime utilizing proton pencil beam scanning technology. During this period, the center operated at steady state, and the RTLS system provided an unbiased and reliable method for collecting treatment time data. This analysis revealed opportunities to enhance consistency in treatment times across gantries and to implement site-specific improvements. Optimizing treatment times can positively impact the patient experience by reducing variability and improving predictability, while also alleviating staff burnout through more streamlined workflows. Furthermore, the data presented here serves as a valuable resource for other centers to inform operational planning and establish benchmarking standards.

## Funding

None.

## CRediT authorship contribution statement

**McConnell:** Conceptualization, Data curation, Formal analysis, Methodology, Writing – original draft. **Wroe:** Conceptualization, Writing – review and editing. **Valladares:** Data curation, Writing – review and editing. **LeGrand:** Data curation, Writing – review and editing. **Ramos:** Data curation, Writing – review and editing. **Chuong:** Writing – review and editing. **Mehta:** Writing – review and editing. **Gutierrez:** Conceptualization, Writing – review and editing, Supervision.

## Declaration of Competing Interest

The authors declare the following financial interests/personal relationships which may be considered as potential competing interests: Michael Chuong Minesh Mehta reports a relationship with Proton Collaborative Group Board of Directors that includes: board membership. Minesh Mehta reports a relationship with NAPT that includes: board membership. Minesh Mehta reports a relationship with NRG Oncology Brain Tumor Committee Chair that includes: board membership. Minesh Mehta reports a relationship with NRG Oncology that includes: board membership. Minesh Mehta reports a relationship with Mevion Medical Systems that includes: board membership. Michael Chuong reports a relationship with Particle Therapy Cooperative Group that includes: board membership. Michael Chuong reports a relationship with International Journal of Radiation Oncology Biology Physics that includes: board membership. Alonso Gutierrez reports a relationship with IBA, Inc - Clinical Advisory Board that includes: board membership. Minesh Mehta has patent pending to Minesh Mehta. Consulting Fees - Minesh Mehta - Telix, Kazia, Novocure, AIQ, GT Medical Technologies Honoraria - Alonso Gutierrez - IBA, ZapX Surgical, Elekta AB, ViewRay, CQ Medical, RadFormation Honoraria - Andrew Wroe - IBA, CQ Medical. If there are other authors, they declare that they have no known competing financial interests or personal relationships that could have appeared to influence the work reported in this paper.
